# From agricultural waste to functional gels: Mulberry pomace-derived carboxymethyl cellulose synergistically enhances calcium-mediated peanut protein isolate gelation

**DOI:** 10.1016/j.fochx.2026.104676

**Published:** 2026-10-24

**Authors:** Yuanfei Ge, Dan Hu, Yujie Sun, Yuqing Tong, Hanxiao Shi, Chuyan Wang

**Affiliations:** a School of Life and Health Sciences, Environmental Engineering, Hefei University, Hefei 230601, China; b State Key Laboratory of Dairy Biotechnology, Key Laboratory of Functional Dairy Products Processing, Ministry of Agriculture and Rural Affairs, Shanghai Engineering Research Center of Dairy Biotechnology, Dairy Research Institute, Bright Dairy and Food Co. Ltd., Shanghai 200436, China; c Key Laboratory of Berry Processing and Resource Comprehensive Utilization, Hefei University, Hefei 230601, China

**Keywords:** Peanut protein isolate, Mulberry pomace-derived carboxymethyl cellulose, Gel performance, Molecular conformation, Gelation mechanism

## Abstract

This study aimed to develop a sustainable functional ingredient from mulberry pomace-derived carboxymethyl cellulose (MPCMC) and elucidate its synergistic role with Ca^2+^ in regulating peanut protein isolate (PPI) gelation. MPCMC was prepared and its effects on PPI gel properties and underlying mechanisms were systematically investigated through multiscale characterization, intermolecular force analysis, correlation analysis, and molecular docking simulation. Results showed that 1.5% MPCMC plus 12 mM Ca^2+^ increased PPI gel strength from 52.47 g to 142.89 g and water holding capacity from 72.21% to 85.49%, with enhanced viscoelasticity and network compactness. Further analyses revealed that Ca^2+^-mediated ionic interactions played an important role in network stabilization, while MPCMC contributed through hydrogen bonding, hydrophobic interactions, and structural filling effects. Molecular docking further supported the potential binding interactions among MPCMC, Ca^2+^, and PPI. These findings establishe a molecular-to-macroscopic relationship for MPCMC and Ca^2+^ regulate PPI gelation, and support mulberry pomace valorization.

## Highlights

• MPCMC and Ca^2+^ synergistically enhanced peanut protein isolate gelation.
• Gelation performance and continuous network were optimal at 1.5% MPCMC and 12 mM Ca^2+^.
• Ca^2+^-mediated ionic bridging and hydrophobic interactions were the principal force for gel reinforcement.
• Findings support mulberry pomace valorization for high-performance plant protein gels.

## 1 Introduction

Plant proteins have attracted considerable attention as sustainable alternatives to animal-derived proteins for application in structured foods, plant-based meat analogues, and functional foods. Gelation is a critical functional attribute of plant proteins, as it directly governs the texture, stability, sensory quality, and processing suitability of food systems. Peanut protein isolate (PPI), as a valuable protein resource obtained from peanut-processing by-products, possesses advantages including high protein content, balanced nutritional composition, low cost, and wide availability (Ma et al., 2026; Peng et al., 2026). These characteristics make PPI a promising candidate for the development of plant-based gel foods. However, compared with animal-derived proteins and some other plant proteins, PPI generally exhibits insufficient gel strength, loose network structures, and limited WHC during heat-induced gelation (Chen et al., 2025; Li et al., 2020; Wang et al., 2026). These structural limitations may result in poor texture, water migration, and instability during processing and storage, thereby restricting the further application of PPI in high-quality plant-based gel products. Therefore, developing effective, safe, and sustainable strategies to improve PPI gelation performance has become an important scientific challenge in the field of plant protein processing.

The formation of protein gels is a complex multistep process involving protein conformational unfolding, molecular aggregation, and network formation, with the resulting gel properties governed by the coordinated balance of multiple intermolecular forces (Ke & Pang, 2026; Zhang, Liang, et al., 2026; Zhang, Zhang, et al., 2026). Growing evidence demonstrated that polysaccharides can strengthen protein gel structures by regulating the extent and pathway of protein aggregation (Ding et al., 2025; Qian et al., 2026). Undoubtedly, such regulatory effects are highly dependent on the source, molecular structure, and compatibility of the polysaccharides itself (Ullah et al., 2025). Carboxymethyl cellulose, a representative anionic cellulose derivative, contains abundant carboxyl and hydroxyl groups, which confer high hydrophilicity and favorable biocompatibility. These properties have drawn increasing attention to carboxymethyl cellulose as a structural regulator of protein gels. Previous studies have demonstrated that carboxymethyl cellulose may also promote gel network reconstruction by regulating protein conformation and the distribution of intermolecular forces (Han et al., 2026; Huang et al., 2024; Sabzevari et al., 2025). However, most commercially available CMC is derived from conventional cellulose resources, while the conversion of agricultural processing residues into functional cellulose derivatives represents a more sustainable strategy aligned with the principles of circular bioeconomy. Mulberry pomace, a major by-product generated during mulberry juice production and processing, contains abundant cellulose and dietary fiber components but is commonly underutilized or discarded, resulting in inefficient resource utilization. Therefore, converting mulberry pomace into mulberry pomace-derived carboxymethyl cellulose (MPCMC) may provide a promising approach for developing sustainable food-grade functional ingredients. Compared with commercial carboxymethyl cellulose, MPCMC derived from agricultural residues may offer advantages in resource sustainability and environmental benefits, while its unique structural characteristics may contribute to specific interactions with plant proteins.

Although polysaccharide-based regulation of protein gelation has been extensively investigated, several limitations remain unresolved. Previous studies have mainly focused on pectin, alginate, and *Lycium barbarum* polysaccharides in protein systems including soy protein isolate, whey protein isolate, and pea protein isolate (Vinceković et al., 2026; Wang, Su, et al., 2025; Wang, Zhang, et al., 2025; Yang et al., 2026). These studies have demonstrated that polysaccharides can regulate protein gelation through hydrogen bonding, electrostatic bonding, and hydrophobic interactions. However, such single-component regulation is typically highly system-dependent and concentration-sensitive, and it often fails to achieve simultaneous optimization of multiple gel properties. Excessive addition may increase steric hindrance, induce phase separation, and interfere with effective protein cross-linking, ultimately weakening gel structure (Xing et al., 2024). More importantly, most previous studies have primarily evaluated the macroscopic effects of individual polysaccharides, while the molecular mechanisms linking polysaccharide-induced intermolecular interactions with final gel performance remain insufficiently understood. In addition to polysaccharides, food-grade metal ions, especially Ca^2+^, have been widely recognized as important regulators of protein gelation. Ca^2+^ can reduce electrostatic repulsion between protein molecules through charge screening effects and promote molecular association through ionic bridging, thereby facilitating protein aggregation and increasing effective cross-linking points within gel networks (Xiao et al., 2021; Zhang et al., 2024). Therefore, combining Ca^2+^ with MPCMC that provide complementary structural regulation may represent a more effective strategy than using either component alone. Nevertheless, the synergistic effects of agricultural by-product-derived polysaccharides and Ca^2+^ on PPI gelation have rarely been explored. In particular, the mechanisms by which MPCMC and Ca^2+^ cooperatively regulate intermolecular forces, protein conformational changes, and gel network construction remain unclear.

This study aimed to (i) develop a sustainable carboxymethyl cellulose ingredient from mulberry pomace, (ii) evaluate the synergistic effects of MPCMC and Ca^2+^ on the gelation behavior and functional properties of PPI, and (iii) elucidate the molecular mechanisms underlying their regulation of intermolecular interactions, protein conformational transitions, and gel network construction. Distinct PPI-based gel systems were established, and a multiscale analytical strategy integrating structural characterization, intermolecular force analysis, correlation analysis, and molecular docking simulation was employed to reveal the relationship between molecular interactions and macroscopic gel performance. These findings provide a targeted strategy for improving plant protein gel quality while enabling the high-value valorization of mulberry pomace as an agroforestry by-product, in line with the principles of green and sustainable modern food manufacturing.

## 2 Materials and methods

### 2.1 Materials and reagents

Mulberry pomace was supplied by Shangqiu Huiyin Food Co., Ltd. (Henan, China). PPI (protein content ≥80%) was purchased from Shanghai Yuanye Biotechnology Co., Ltd. (Shanghai, China). Chloroacetic acid (CAS 79–11-8) and sodium chlorite (CAS 7758-19-2) were provided by Shanghai Macklin Biochemical Co. Ltd. (Shanghai, China). Calcium chloride anhydrous (CAS 10043–52-4) was obtained from Sinopharm Chemical Reagent Co. Ltd. (Shanghai, China). All other reagents were of analytical grade and used without purification.

### 2.2 Mulberry pomace cellulose (MPC) preparation

MPC preparation was performed according to the method reported by Dai and Huang (2016), and with some modifications. Approximately 25 g of mulberry pomace powder was suspended in 500 mL of deionized water and heated in a water bath at 80 °C for 2 h. After filtration, the extraction procedure was repeated once under the same conditions. The obtained insoluble fraction was subsequently treated with 500 mL of 7.5% sodium chlorite solution (*w*/*v*, pH 4.0) and stirred at 75 °C for 2 h. The reaction mixture was sequentially washed with deionized water and 95% (*v*/v) ethanol until the pH reached 7.0, followed by drying at 50 °C for 24 h and subsequent pulverization. The pulverized sample was then immersed in 10% (*w*/*v*) NaOH solution and agitated on a shaker at 25 °C for 12 h, followed by another wash to achieve a pH 7.0. Finally, the product was freeze-dried at −50 °C for 24 h to obtain MPC.

### 2.3 MPCMC preparation

MPCMC was synthesized following the procedure reported by Zheng et al. (2025), and with slight modifications. MPC powder (5 g) was suspended in 100 mL isopropanol and allowed to swell under stirring at 25 °C for 0.5 h. An 11 mL NaOH solution (45%, *w*/*v*) was slowly added under stirring, followed by alkalization at 28 °C for 1.5 h. Subsequently, 17 mL isopropanol solution containing chloroacetic acid (6.0 g) and NaOH (0.5 g) was added dropwise. The reaction mixture was heated to 60 °C and maintained under etherification conditions for 4 h. After completion of the reaction, the mixture was neutralized to pH 7.0 with 10% (*v*/v) glacial acetic acid. The precipitate was successively washed with 80% methanol and absolute methanol, and then dried at 50 °C to obtain MPCMC. The degree of substitution of MPCMC was determined as 0.92 ± 0.07 according to a previously reported method (Dai & Huang, 2017).

### 2.4 PPI-based gel samples preparation

PPI powder was dispersed in deionized water, and the pH was adjusted to 7.0. The dispersion was magnetically stirred (600*g*) at 25 °C for 2 h, and then fully hydrated at 4 °C for 12 h to obtain a 20% (*w*/*v*) stock solution. MPCMC stock solutions with concentrations of 0%, 2%, 4%, 6%, and 8% (*w*/*v*) were prepared following the similar procedure.

The PPI and MPCMC stock solution or CaCl_2_ were blended at 25 °C, and the pH was subsequently adjusted to 7.0. In all formulations, the final PPI concentration was maintained at 15% (*w*/*v*), whereas the final MPCMC concentration was set to 0%, 0.5%, 1%, 1.5%, and 2% (*w*/*v*), and the Ca^2+^ concentration was set at 0, 4, 8, 12, and 16 mM, respectively. After magnetic stirring (600 ×g, 30 min), the mixtures were transferred to a 95 °C water bath and heated for 30 min, rapidly cooled, and then incubated at 4 °C for 12 h to complete gelation. The optimal concentrations of MPCMC and Ca^2+^ were determined based on gel strength as the primary evaluation criterion, and the highest gel strength was selected as the optimal condition for subsequent experiments. The gel samples prepared under these optimized conditions were designated as PPI/Ca^2+^, MPCMC/PPI, and MPCMC/PPI/Ca^2+^, respectively. A 15% (*w*/*v*) PPI gel served as the control.

### 2.5 FTIR and XRD measurement

FTIR spectra of MPC, MPCMC, and freeze-dried gels were recorded on a FTIR spectrometer (Nicolet IS20, Thermo Scientific, Waltham, MA, USA), with a resolution of 2 cm^−1^ from 4000 to 650 cm^−1^. Deconvolution of the amide I band (1700–1600 cm^−1^) in the obtained spectra was performed using PeakFit software (Systat Software Inc., San Jose, CA, USA) to calculate the relative proportions of protein secondary structures (Ge et al., 2026).

XRD patterns of MPC, MPCMC, and freeze-dried gel samples were recorded using an X-ray diffractometer (ZSX Primus II, Rigaku, Akishima, Tokyo, Japan). The XRD analysis was performed at 40 kV and 40 mA, with a scanning rate of 5°/min over a 2θ range of 5–60°.

### 2.6 Scanning electron microscopy (SEM) observation

The samples were mounted onto specimen stubs using conductive adhesive tape, and sprayed with gold. The micromorphology was imaged using a SEM (EM-30^+^, COXEM, Daejeon, South Korea). Image J software (NIH, Bethesda, MD, USA) was applied to quantitatively determine the porosity of gel samples.

### 2.7 Thermal measurement

The thermal stability of freeze-dried samples was evaluated using a thermogravimetric analyzer (Q500, TA Instruments, New Castle, USA). The measurements were performed under a nitrogen atmosphere at a heating rate of 10 °C/min from 30 °C to 800 °C, and the continuous mass change curve was recorded as a function of temperature.

### 2.8 Gel strength and water holding capacity (WHC) determination

Gel strength was measured by a single-compression test on a texture analyzer (TA-XT plus, Stable Microsystems Co., UK), equipped with a P/0.5 probe. The pre-test, test, and post-test speeds were set to 2.0, 1.0, and 2.0 mm/s, respectively. The compression strain was 40% of the original sample height, with a trigger force of 5.0 g. All samples were equilibrated at 25 °C for 30 min prior to measurement. Gel strength was defined as the maximum force recorded on the force-deformation curve during compression.

The WHC of gel samples was determined using a centrifugation method (Ge et al., 2025). The weight of the centrifuge tube containing the fresh gel sample was recorded as W_0_. After centrifugation at 4 °C (10,000 ×g, 15 min), the separated water was discarded, and the inner wall of the tube was carefully blotted with filter paper to remove residual water droplets. The tube containing the gel was then reweighed, and recorded as W_1_. The mass of the empty centrifuge tube (W_c_) was also recorded separately. WHC was calculated according to Eq. (1).

(1) $$\mathrm{WHC}=\left(W_{1}-W_{c}\right)/\left(W_{0}-W_{c}\right)\times 100\%$$

### 2.9 Atomic force microscopy (AFM) observation

The microstructural features and average roughness parameters of gel samples were measured by AFM (Dimenson ICON, Bruker, Karlsruhe, Germany). Prior to imaging, a small amount of gel sample was uniformly dispersed in deionized water, and the suspension was diluted to a final concentration of 0.01 mg/mL. The prepared suspensions were then deposited onto the clean mica slides, and dried at 25 °C for 24 h to obtain uniformly deposited specimens without obvious aggregation. All AFM images were acquired in tapping mode using standard silicon cantilevers under ambient conditions at 25 °C, with a scanning frequency of 1 Hz.

### 2.10 Low field nuclear magnetic resonance (LF-NMR) and rheological behavior

The transverse relaxation time (T_2_) and water distribution of the different gel samples were determined using a LF-NMR analyzer (MesoMR23-060H-I, Niumag Co., Shanghai, China) to evaluate water mobility within the gel systems (Xu et al., 2025). Prior to measurement, each sample (3.0 g) was placed in a dedicated NMR tube and sealed to prevent water evaporation. All tests were performed at 25 °C, and the Carr-Purcell-Meiboom-Gill pulse sequence was employed to acquire proton relaxation signals. The main parameters were set as follows: spectral width, 200 kHz; repetition time, 5000 ms; number of echoes, 20000; and echo time, 0.3 ms.

A rheometer (RH-20, Bosin Tech, Shanghai, China) equipped with a 50 mm parallel-plate was used to perform frequency sweeps over an angular frequency range of 0.1–100 rad/s. The storage modulus (G′), loss modulus (G′′), and loss tangent (tan δ = G′′ / G′) were recorded as functions of frequency. The gap between the plates was set to 1 mm, and the temperature was maintained at 25 °C throughout the measurements. Furthermore, the frequency dependence of G′ and G″ was fitted using a power-law model, and as follows:

(2) $$G^{\prime }\left(\omega \right)=K^{\prime }\;\omega^{n\prime }$$

(3) $$G^{\prime \prime }\left(\omega \right)=K^{\prime \prime }\;\omega^{n\prime \prime }$$

where K′ and K″ are power law constants, and n' and n″ represent the frequency dependence of G′ and G″, respectively.

### 2.11 Intrinsic fluorescence spectroscopy measurement

Intrinsic fluorescence spectra of proteins (1 mg/mL) in the gel samples were recorded using a fluorescence spectrometer (FLS980, Edinburgh Instrumens, Livingston, UK), equipped with a 1 cm quartz cuvette. The spectra were scanned over 300–450 nm at 25 °C with an excitation wavelength of 280 nm. The excitation and emission slit widths were both set to 5 nm.

### 2.12 Protein solubility and molecular simulation

The molecular interactions involved in the gel network were evaluated by measuring protein solubility in a series of selective solvents. Accurately weighed gel samples (1.0 g) were mixed with 10 mL of the solvents listed in Table 1, incubated at 25 °C for 1 h, followed by centrifugation (10,000*g*, 15 min). The protein content of the supernatant was determined by the Bradford method. S1 served as the control, and the relative contributions of ionic bonds (S2 vs. S1), hydrogen bonds (S3 vs. S2), hydrophobic interactions (S4 vs. S3), and disulfide bonds (S5 vs. S4) were calculated based on differences in solubility (Lin, Mao, et al., 2026; Lin, Xu, et al., 2026).

**Table 1:** Composition of different solvents.

Solvents	0.05 M NaCl	0.6 M NaCl	1.5 M Urea	8 M Urea	0.5 M β-mercaptoethanol
S1	+	−	−	−	−
S2	−	+	−	−	−
S3	−	+	+	−	−
S4	−	+	−	+	−
S5	−	+	−	+	+

Molecular docking simulations were performed to elucidate the potential interaction modes between Ca^2+^, MPCMC, and PPI at the molecular level. Peanut 7S globulin, the core trimeric subunit of arachin, was selected as the protein receptor, and its crystal structure was retrieved from the PDB (ID: 3S7I) database (http://www.rcsb.org) (Yan et al., 2026). Water molecules and heteroatoms were removed using PyMol to obtain the purified structure. The repeating unit of carboxymethyl cellulose (CID: 24748) was used as the small-molecule ligand. Its three-dimensional structure was downloaded from PubChem database (https://pubchem.ncbi.nlm.nih.gov), and optimized by energy minimization to obtain a stable conformation. Molecular docking calculations were performed with AutoDock 1.5.7. The docking grid was set to cover the putative binding region of the protein. A global conformational search was conducted using the Lamarckian genetic algorithm, with all other parameters were kept at their default values. The docking pose with the lowest binding energy and most stable conformation was selected for interaction analysis.

### 2.13 Statistical analysis

All experiments were performed in triplicate, and the data were expressed as mean ± standard deviation. Statistical analyses were carried out using IBM SPSS Statistics 26.0 (IBM Software, Armonk, NY, USA). One-way analysis of variance (ANOVA) followed by the least significant difference (LSD) multiple comparison test was employed to evaluate differences among groups, with a *p* < 0.05 considered statistically significant. In addition, Pearson correlation analysis was conducted to investigate the relationships among gel strength, WHC, porosity, power-law rheological parameters, and molecular interactions within the gel networks.

## 3 Results and discussion

### 3.1 MPCMC characterization

Carboxymethyl cellulose is a safe and cost-effective anionic cellulose derivative with broad potential for regulating protein gel structures, constructing delivery vehicles, and developing high-performance plant-based edible gels. Fig. 1A schematically illustrates the main preparation route from mulberry pomace to MPCMC. Mulberry pomace powder was subjected to thermal treatment, delignification, and alkalization to remove impurities and obtain MPC. Subsequently, MPC underwent swelling and etherification under alkaline conditions, followed by washing and drying, to obtain MPCMC. The FTIR spectra of MPC and MPCMC are presented in Fig. 1B. The absorption bands at 3500–3200 cm^−1^, 2900 cm^−1^, 1321 cm^−1^, and 1051 cm^−1^ were attributed to O—H stretching, C—H stretching, O—H bending, and C—O—C stretching vibrations, respectively, while the peak at 894 cm^−1^ corresponds to β—C—H deformation vibrations. The presence of these signals in both samples indicated that carboxymethylation did not destroy the cellulose backbone (Xu et al., 2024). In comparison with MPC, two intense absorption bands at 1600 cm^−1^ and 1420 cm^−1^ were observed for MPCMC, which separately corresponded to the asymmetric and symmetric stretching vibrations of carboxymethyl anion –COO^−^. This result confirmed that etherification successfully grafted anionic carboxymethyl groups onto the hydroxyl sites of cellulose glucopyranose units, yielding MPCMC (Zheng et al., 2025). XRD patterns (Fig. 1C) revealed that MPC exhibited typical cellulose I diffraction peaks at 2θ = 14.6°, 22.0°, and 34.5°, indicating that it retained a partially crystalline cellulose structure. After carboxymethylation, the characteristic diffraction peaks of MPCMC were significantly weakened and broadened, with the peaks at 2θ = 14.6° and 34.5°disappeared, and a weaker peak appeared at approximately 2θ = 20.8°. These changes suggest that alkalization and etherification processes weakened the ordered packing of cellulose molecular chains and partially disrupted the crystalline domains. This phenomenon may be related to the fact that the introduction of carboxymethyl groups, which perturbed the interchain hydrogen-bonding network of cellulose and increased the proportion of amorphous regions (Dai & Huang, 2017; Zheng et al., 2025). The micromorphology of MPC and MPCMC were observed by SEM (Fig. 1D). MPC exhibited a relatively flat and smooth surface, with compact, rigid, rod-like fibrous structures. After carboxymethylation, MPCMC underwent pronounced swelling and thickening, accompanied by abundant longitudinal wrinkles, protrusions, and grooves, which resulted in markedly increased surface roughness. This altered morphology may endow MPCMC with enhanced WHC and interfacial binding activity. These observations are consistent with the findings reported by Sethi et al. (2022) and Zheng et al. (2025). Fig. 1E shows that both MPC and MPCMC underwent a three-stage thermal degradation process. In the 30–200 °C stage, mass losses of 7.63% and 12.33% were observed for MPC and MPCMC, respectively, primarily due to moisture evaporation, indicating the enhanced capacity of MPCMC to bind hydrophilic water. The 200–500 °C interval corresponded to rapid degradation, during which MPC and MPCMC exhibited mass losses of 63.96% and 47.21%, respectively, while the maximum thermal degradation temperature of MPCMC shifted to a lower temperature. This result indicated that carboxymethylation weakened the interchain hydrogen-bonding interactions within cellulose, thereby reducing the activation energy required for thermal degradation (Zhang et al., 2025). During the 500–800 °C stage, MPCMC retained a higher final residual mass than MPC. Overall, MPCMC exhibited sufficient thermal stability to meet the conventional thermal processing requirements of plant-based edible gels.

**Fig. 1:**
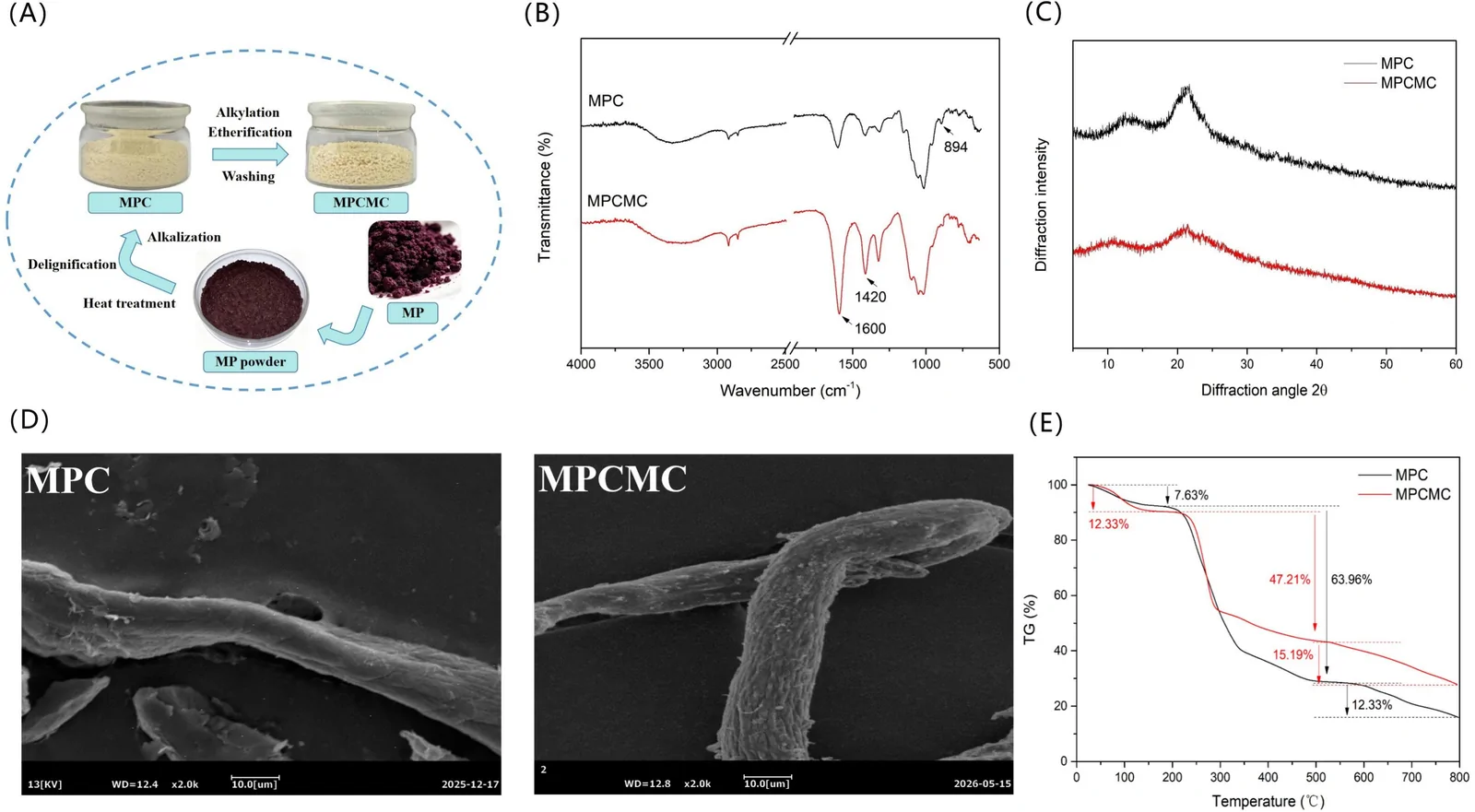
(A) Preparation procedure for MPCMC. (B) FTIR spectra, (C) XRD patterns, (D) SEM images, and (E) TG curves of MPC, MPCMC.

### 3.2 Gel strength analysis

Gel strength is a key quantitative indicator of the textural quality of protein gels. As shown in Fig. 2A, the strength of PPI gel exhibited a clear Ca^2+^ concentration-dependent response, increasing from 52.47 ± 2.50 g to 100.17 ± 1.18 g. This enhancement can be attributed to the ability of Ca^2+^ to shield negative charges on the PPI molecular surface, which reduces the electrostatic repulsion between protein molecules (Zhang et al., 2024). Meanwhile, Ca^2+^ may promote protein aggregation and cross-linking, thereby ensuring the compactness of the gel network. However, when the Ca^2+^ concentration was further increased to 16 mM, the strength of PPI gel decreased to 75.38 ± 1.19 g. It can be inferred that excessive Ca^2+^ may induce rapid or localized overaggregation of proteins, resulting in a coarse, loose, and heterogeneous gel network (Jiang et al., 2026). A similar dose-dependent pattern was also observed for the effect of MPCMC on PPI gel strength. Fig. 2B shows that MPCMC incorporation significantly improved PPI gel strength, indicating that MPCMC not only served as a polysaccharide filler phase, but also contributed to the construction of the composite gel network through intermolecular interactions mediated by hydrophilic functional groups, such as carboxymethyl and hydroxyl groups (Liu et al., 2026; Zhang, Liang, et al., 2026; Zhang, Zhang, et al., 2026). Notably, excessive MPCMC may induce steric hindrance or thermodynamic incompatibility, thereby impeding the ordered aggregation of PPI molecules and weakening the structural uniformity and mechanical strength of the gel, as evidenced by the decrease in gel strength from 81.50 ± 4.57 g to 73.05 ± 3.06 g. Therefore, the effect of different Ca^2+^ concentrations on MPCMC/PPI gel strength in the presence of 1.5% MPCMC was further investigated, as shown in Fig. 2C. Compared with the individual addition of Ca^2+^ or MPCMC, their combined incorporation further modulated and reinforced PPI gel strength, indicating a pronounced synergistic effect between the two components. At an appropriate Ca^2+^ concentration (12 mM), the gel strength reached the highest value (142.89 ± 3.18 g) among all groups, substantially exceeding that of any single-component system. This synergistic effect may originate from the fact that Ca^2+^ improves PPI gelation and participates in the interaction between MPCMC and PPI, thereby promoting the formation of a more stable gel network. Based on the above results, the dose-response relationship for the synergistic reinforcement of PPI gels by MPCMC and Ca^2+^ was clarified, and 1.5% MPCMC and 12 mM Ca^2+^ were identified as the preferred formulation conditions. The inverted macroscopic images (Fig. 2D) show that PPI gel, PPI/Ca^2+^, MPCMC/PPI, and MPCMC/PPI/Ca^2+^ all completed gelation, with no liquid flow or structural collapse after inversion. Among these samples, MPCMC/PPI/Ca^2+^ exhibited superior self-supporting capacity.

**Fig. 2:**
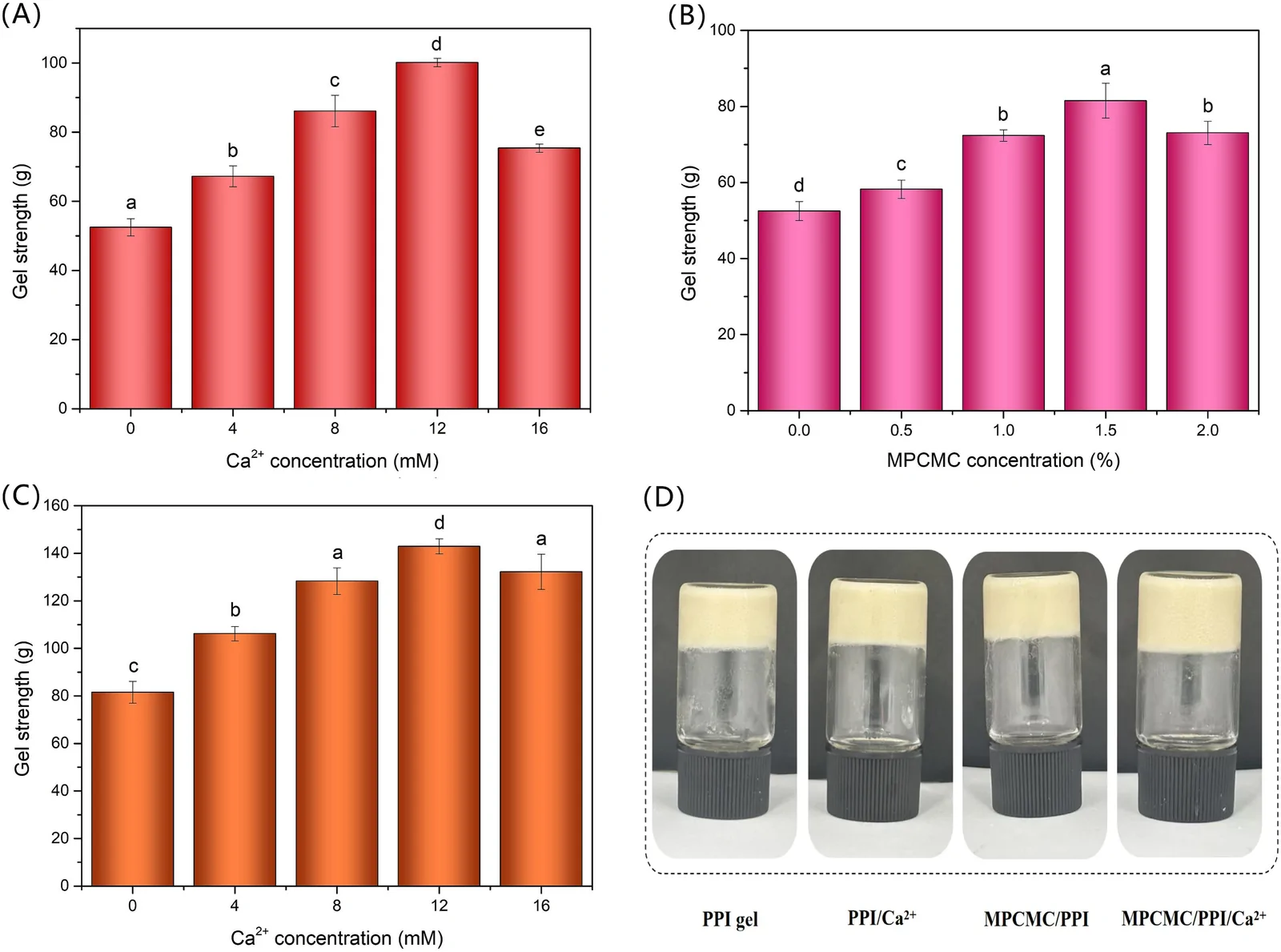
(A) Effect of different Ca^2+^ and (B) MPCMC concentrations on PPI gel strength. (C) Effect of different Ca^2+^ concentrations on the gel strength of MPCMC/PPI containing 1.5% MPCMC. (D) Images of samples prepared at the optimal Ca^2+^ and/or MPCMC concentrations. Values with the different letter (a–e) indicate significant differences (*p* < 0.05).

### 3.3 Thermal analysis

TG curves are widely used to evaluate the mass loss behavior and thermal stability of gel systems during heating. As shown in Fig. 3, all samples exhibited a typical two-stage thermal degradation. The mass loss in the first stage (30–200 °C) was mainly attributed to the evaporation of bound water (Yang et al., 2025). The mass losses of the PPI gel and PPI/Ca^2+^ were both 3.44%. In contrast, the mass loss of MPCMC/PPI increased to 4.99%, indicating that MPCMC enhanced the water-binding and WHC of the system. This enhancement may be related to the abundant hydroxyl and carboxymethyl groups along the MPCMC molecular chains. Notably, the mass loss of the MPCMC/PPI/Ca^2+^ in this stage was 4.22%, indicating that Ca^2+^ addition may promote the formation of a more stable network between MPCMC and PPI, and shift water from a relatively loosely bound state to a more tightly bound state. Similar ion-induced water state transitions have also been observed in Ca^2+^-regulated soy protein isolate and cellulose nanocrystal composite gel systems (Zhang et al., 2024). The second stage occurred between 200 and 800 °C, and mainly involved the cleavage of protein peptide chains and degradation of the carboxymethyl cellulose backbone (Tong et al., 2024). The onset degradation temperatures of the PPI gel, PPI/Ca^2+^, MPCMC/PPI, and MPCMC/PPI/Ca^2+^ were 226.75 °C, 231.95 °C, 228.93 °C, and 233.71 °C, respectively. Compared with the PPI gel, MPCMC/PPI showed a reduced mass loss of 69.88% during the 200–800 °C, indicating that MPCMC mitigated thermal mass loss at elevated temperatures. More importantly, MPCMC/PPI/Ca^2+^ exhibited the lowest mass loss in this stage, at only 68.43%, further demonstrating that the synergistic effect of Ca^2+^ and MPCMC enhanced the structural stability and residual mass of the gel system under high-temperature conditions. These findings provide important support for its application in thermally processed plant-protein gel foods.

**Fig. 3:**
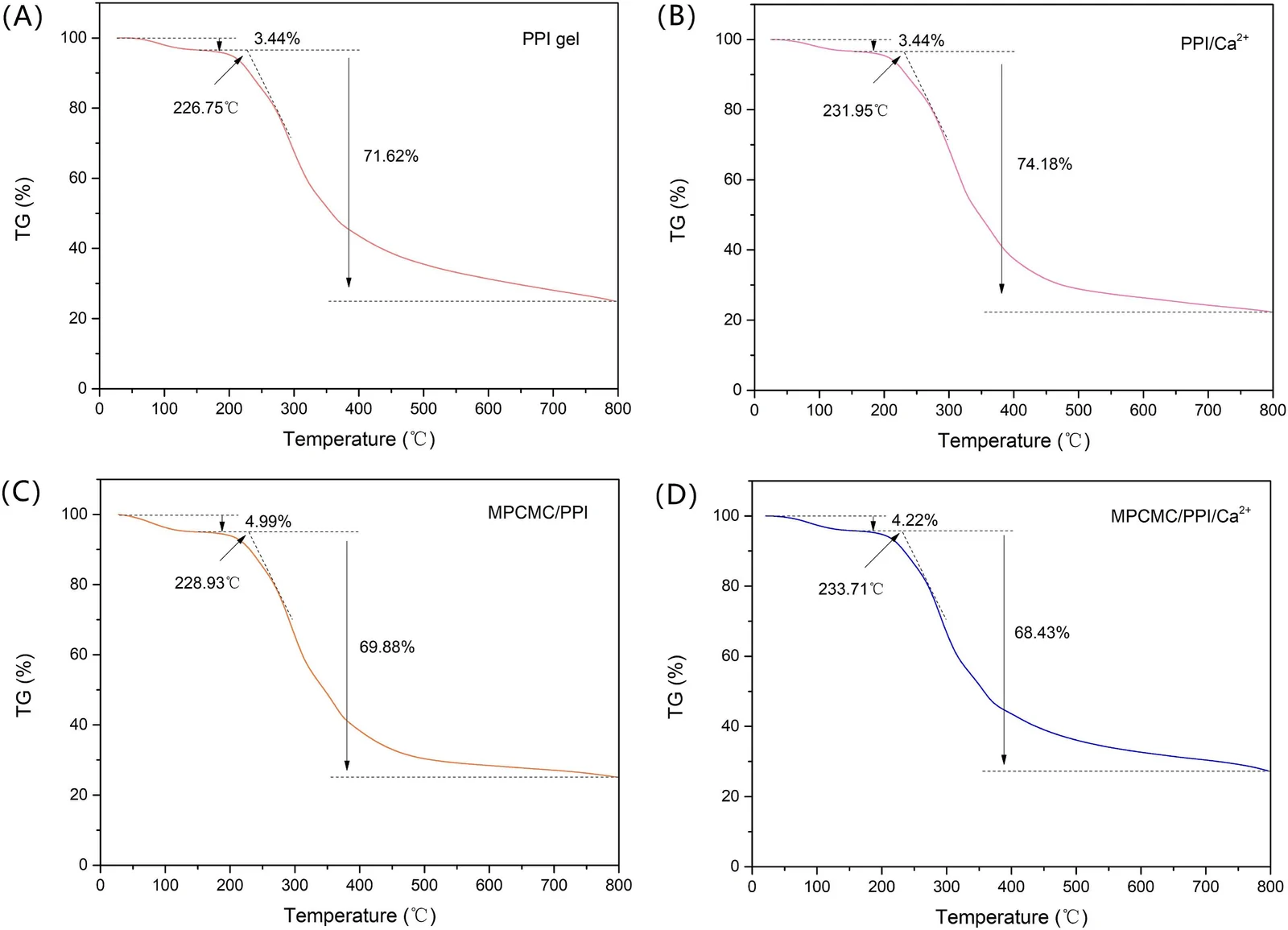
TG curves of (A) the PPI gel, (B) PPI/Ca^2+^, (C) MPCMC/PPI, and (D) MPCMC/PPI/Ca^2+^.

### 3.4 WHC, LF-NMR, and rheological behavior analysis

WHC reflects the capacity of the gel network to physically entrap water and directly indicates network compactness and homogeneity. As shown in Fig. 4A, the PPI gel exhibited a relatively limited ability to immobilize water, with a WHC of 72.21 ± 1.95%. The incorporation of Ca^2+^ alone significantly increased the WHC to 80.49 ± 2.51%. As discussed in the gel strength analysis, this improvement is likely attributable to charge shielding by Ca^2+^ and its promotion of PPI molecular aggregation, which collectively enhanced the capacity of the gel network to retain water. In contrast, the addition of MPCMC alone produced a more modest increase in WHC, corresponding to an increase of 6.9%. The abundant hydroxyl and carboxymethyl groups along the MPCMC chains conferred strong hydrophilicity, while the physical filling of voids within the protein network further contributed to improved water retention. Notably, MPCMC/PPI/Ca^2+^ exhibited the highest WHC (85.49 ± 3.64%), substantially exceeding that reported for certain cellulose nanocrystal/Ca^2+^-reinforced protein gel systems. For example, Zhang et al. (2024) found that the combined incorporation of cellulose nanocrystals and Ca^2+^ did not yield a pronounced synergistic enhancement in WHC, whereas MPCMC and Ca^2+^ exhibited a marked synergistic effect in the present study. These results indicate that the synergistic action of Ca^2+^ and MPCMC generated an efficient water-entrapment network.

**Fig. 4:**
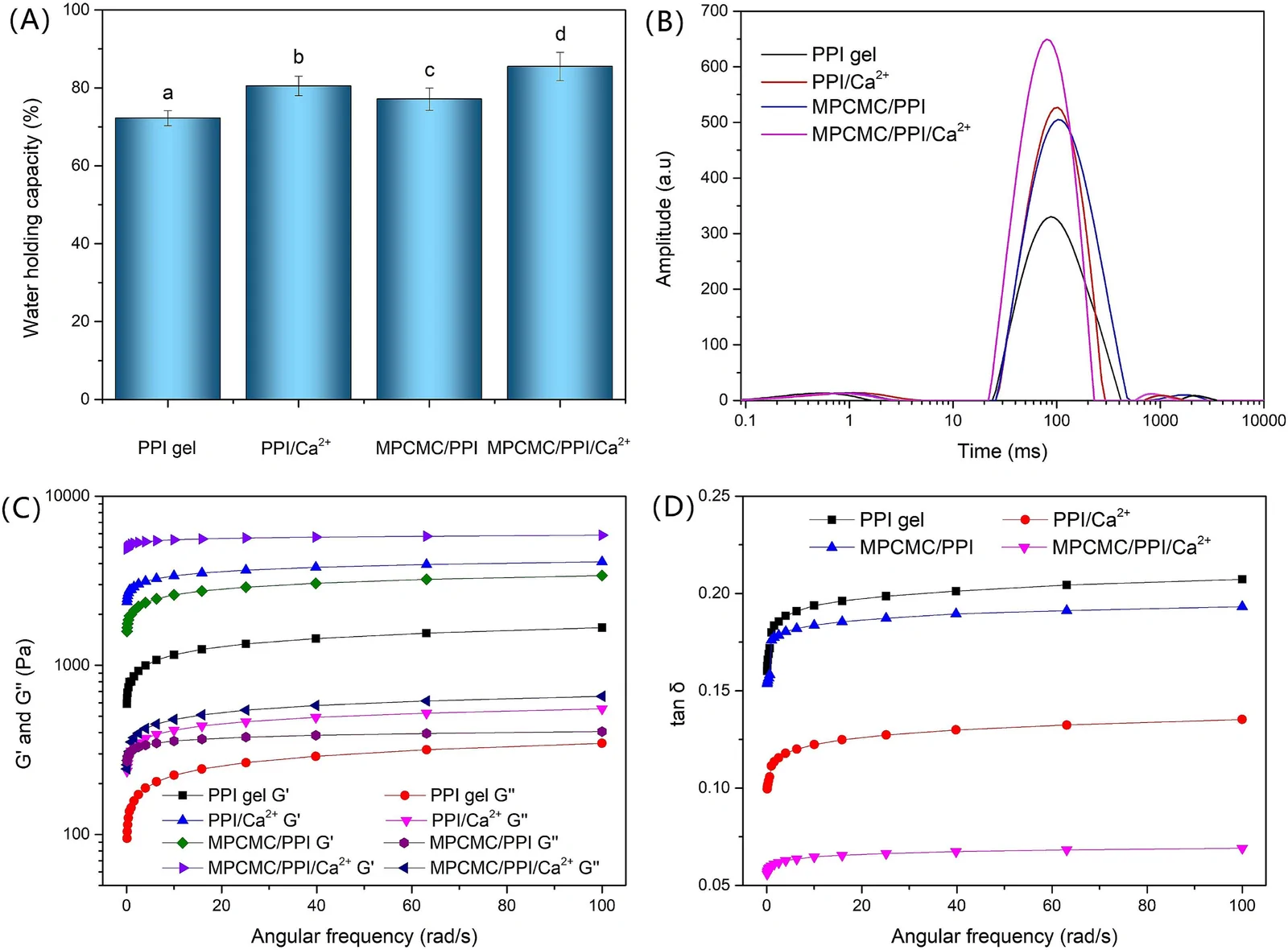
(A) WHC, (B) relaxation time curves, and (C, D) rheological behavior of the PPI gel, PPI/Ca^2+^, MPCMC/PPI, and MPCMC/PPI/Ca^2+^. Values with the different letter (a–d) indicate significant differences (*p* < 0.05).

Fig. 4B and Table S1 further characterize the water state and mobility in the different gel systems using LF-NMR T_2_ relaxation analysis. The PPI gel, PPI/Ca^2+^, MPCMC/PPI, and MPCMC/PPI/Ca^2+^ exhibited similar relaxation profiles, each displaying three relaxation peaks corresponding to bound water (T_21_), immobilized water (T_22_), and free water (T_23_). In the PPI gel, the network imposed only limited constraints on water mobility. Although water was predominantly present as immobilized water, a measurable fraction of free water remained. The LF-NMR T_2_ relaxation time is closely associated with the degree of interaction between water molecules and the gel matrix (Hu et al., 2025). The incorporation of Ca^2+^ altered water mobility, significantly reducing T_23_ in PPI/Ca^2+^ to 1075.28 ± 47.36 ms, while P_23_ decreased from 2.57 ± 0.06% to 1.53 ± 0.15%. Concurrently, P_22_ increased to 96.29 ± 0.77%, indicating that a larger proportion of water was immobilized within the gel network. Compared with PPI gel, MPCMC/PPI also showed a pronounced reduction in the proportion of free water, with P_23_ decreasing to only 0.82 ± 0.03%. Although the two additives may act through distinct mechanisms within the gel matrix, both tended to restrict water migration. The MPCMC/PPI/Ca^2+^ exhibited the lowest free-water relaxation time and free water proportion. Specifically, T_23_ decreased from 2125.67 ± 158.32 to 792.21 ± 75.93 ms, accompanied by a reduction in P_23_ to 0.69 ± 0.09%. These results further demonstrate that the synergistic effect of Ca^2+^ and MPCMC enabled water to be more stably entrapped within the gel network. In addition, the dominant P_22_ showed no significant differences among the groups, which precisely indicates that Ca^2+^ and MPCMC, whether alone or in combination, did not markedly alter the total amount of immobilized water in the gel; rather, the observed changes mainly originated from alterations in the bound-water and free-water fractions. The above results are also highly consistent with the WHC.

Fig. 4C records the dynamic rheological behavior of PPI gel, PPI/Ca^2+^, MPCMC/PPI, and MPCMC/PPI/Ca^2+^, as reflected by the frequency dependence of G′ and G′′. All samples exhibited G′ values substantially higher than G′′ throughout the tested angular frequency range, indicating the formation of continuous gel networks dominated by elastic behavior. The variations in G′ and G′′ further revealed that the incorporation of Ca^2+^ and MPCMC markedly reinforced the characteristics of the gel networks. In particular, when Ca^2+^ and MPCMC were present simultaneously, both G′ and G′′ reached their maximum values in the MPCMC/PPI/Ca^2+^, demonstrating the strongest elastic response and greatest network strength. This structural reinforcement likely originates from the synergistic contributions of Ca^2+^-mediated ionic cross-linking and intermolecular interactions between MPCMC and PPI, thereby ensuring the integrity and stability of the gel network (Lv et al., 2025; Xie et al., 2025). Tan δ (Fig. 4D) reflects the relative contributions of viscous and elastic behavior in gel systems. The tan δ values (0.05–0.20) of all samples were < 1, a characteristic dominated by elastic response over the tested frequency range. Moreover, tan δ exhibited an inverse trend relative to G′ and G′′, indicating that the addition of Ca^2+^ and MPCMC reduced the proportion of viscous dissipation relative to elastic energy storage. Accordingly, the gel systems tended to store deformation energy rather than undergo viscous flow or energy dissipation under external force. Similar phenomena have also been reported for pea protein isolate gelation reinforced by citrus peel cellulose nanofibers (Lin, Mao, et al., 2026; Lin, Xu, et al., 2026).

The power-law model parameters further quantitatively described the angular frequency-dependent behavior of the gel systems, as shown in Table 2. Compared with the PPI gel, the incorporation of Ca^2+^ and MPCMC markedly increased K′ and K′′, and this was especially pronounced for MPCMC/PPI/Ca^2+^, with values of 5210.06 ± 7.54 and 352.11 ± 0.87, respectively. This also further confirmed the gel strength measurement results. It also indicates that this gel maintained a highly elastic network and structural integrity while retaining a certain capacity for energy dissipation, which may contribute to its resistance to structural failure under external stress. The n' and n'′ reflect the sensitivity of the moduli to frequency variation. For all samples, n' (0.020–0.127) and n'′ (0.078–0.180) were < 1, and were evidently affected by the addition of Ca^2+^ and/or MPCMC, consistent with the frequency-dependent changes in G′ and G′′. The high R^2^ values (0.980–0.998) of the fitted models confirmed that the power-law model adequately described the angular-frequency sweep behavior of the different gel systems.

**Table 2:** Power-law function parameters of the PPI gel, PPI/Ca^2+^, MPCMC/PPI, and MPCMC/PPI/Ca^2+^.

Samples	K′	n'	R^2^	K′′	n'′	R^2^
PPI gel	800.52 ± 5.21^a^	0.127 ± 0.003^c^	0.998	144.73 ± 1.25^d^	0.180 ± 0.005^c^	0.998
PPI/Ca^2+^	2797.87 ± 6.89^b^	0.061 ± 0.002^b^	0.997	337.15 ± 0.68^c^	0.078 ± 0.004^d^	0.995
MPCMC/PPI	2002.39 ± 3.91^c^	0.086 ± 0.003^b^	0.997	320.36 ± 0.95^b^	0.116 ± 0.008^a^	0.980
MPCMC/PPI/Ca^2+^	5210.06 ± 7.54^d^	0.020 ± 0.002^a^	0.994	352.11 ± 0.87^a^	0.102 ± 0.006^b^	0.987

Values with the different letter (a–d) indicate significant differences (*p* < 0.05).

### 3.5 FTIR, XRD, and intrinsic fluorescence spectroscopy analysis

Fig. 5A presents the FTIR spectra of the PPI gel, PPI/Ca^2+^, MPCMC/PPI, and MPCMC/PPI/Ca^2+^. All samples exhibited distinct absorption bands at approximately 3275 cm^−1^ and 2922 cm^−1^, assigned to the stretching vibrations of —OH/—NH and C—H, respectively, which are typical signals of the protein and polysaccharide molecular backbones. Compared with the PPI gel, the C=O stretching vibration band red-shifted to 1627 cm^−1^ in both PPI/Ca^2+^ and MPCMC/PPI, and further shifted to 1623 cm^−1^ in MPCMC/PPI/Ca^2+^. This shift may arise from changes in the microenvironment surrounding protein molecules, and the associated alterations in intermolecular interactions and protein conformation (Ge et al., 2026). Although no new absorption bands appeared among the treatment groups, the preservation of similar characteristic peaks indicated that the fundamental functional groups within the gel systems remained unchanged. Nevertheless, these spectral changes suggest that the incorporation of Ca^2+^ and MPCMC likely altered the strength of intermolecular hydrogen bonding and hydrophobic interactions (Chen et al., 2026). The relative contents of protein secondary structures in the different gels were quantified by deconvolution of the amide I band (Fig. 5B). The β-sheet content increased markedly after the incorporation of Ca^2+^ and MPCMC, reaching a peak value of 46.52% in MPCMC/PPI/Ca^2+^. The β-sheet content reflects the extent of intermolecular interactions and the ordering of the gel network. A similar phenomenon has been reported for whey protein isolate gelation induced by Ca^2+^ and cellulose nanocrystals (Xiao et al., 2021). Concurrently, the α-helix and random coil contents decreased from 20.31% and 19.95% to 17.75% and 17.56%, respectively, although these reductions were relatively limited. These changes further confirmed that the transition of disordered peptide chains into ordered β-sheet aggregates provided a molecular basis for gel network densification (Lv et al., 2025; Zhao et al., 2026). The secondary structure results are consistent with the red shift of the FTIR amide I band, providing direct structural evidence for the effects of Ca^2+^ and MPCMC.

**Fig. 5:**
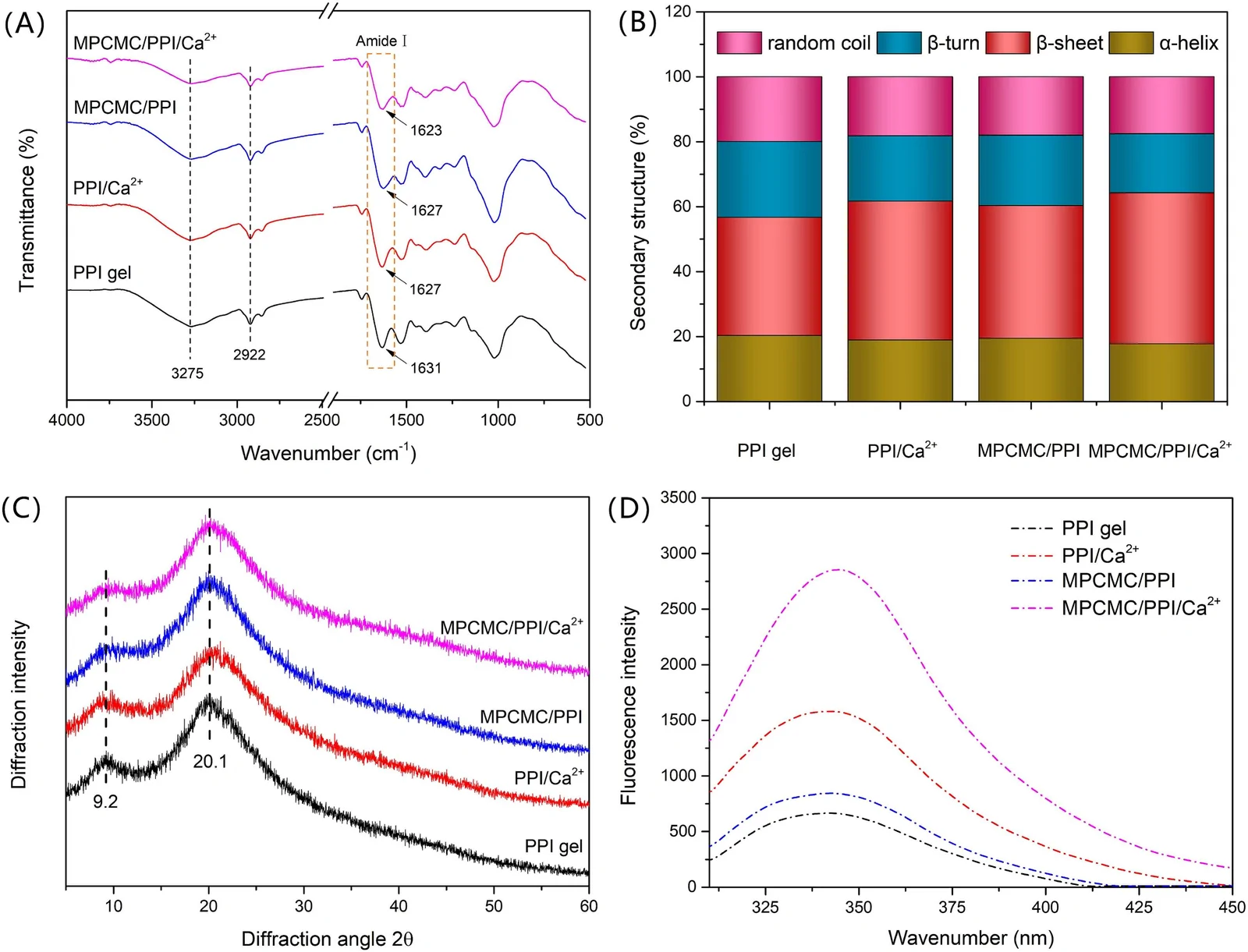
(A) FTIR spectra, (B) secondary structure content, (C) XRD patterns, and (D) intrinsic fluorescence spectra of the PPI gel, PPI/Ca^2+^, MPCMC/PPI, and MPCMC/PPI/Ca^2+^.

XRD patterns (Fig. 5C) were used to evaluate the effects of Ca^2+^ and/or MPCMC on the crystalline characteristics and molecular arrangement of PPI gels. All samples exhibited characteristic diffraction peaks at approximately 2θ = 9.2° and 20.1°, confirming that the PPI gels retained the basic structural features of the protein matrix after different treatments. The absence of newly emerged diffraction peaks indicated that the incorporation of Ca^2+^ and MPCMC did not induce new crystalline phases formation. Compared with the PPI gel, the addition of Ca^2+^ and/or MPCMC progressively reduced the diffraction intensities at approximately 2θ = 9.2° and 20.1°. The original locally ordered structures of PPI were disrupted and that the crystallinity or short-range ordering of the gel matrix decreased. Similar phenomena have been reported by Xu et al. (2025) and Xiao et al. (2021). It should be emphasized that these diffraction features are not uniquely diagnostic of specific protein secondary structures. Rather, they primarily reflect characteristic intermolecular spacing and local packing within the protein matrix. Therefore, the decrease in XRD diffraction intensity does not conflict with the increase in β-sheet content revealed by FTIR analysis. However, the reduced XRD intensity suggested that such ordering occurred primarily at the level of local intermolecular conformations or short-range aggregates rather than developing into a regular crystalline structure. In MPCMC/PPI/Ca^2+^, the diffraction peaks at both positions decreased further, clearly demonstrating that the structural reinforcement did not originate from an increased crystallinity, but rather primarily from enhanced multiple intermolecular interactions within the amorphous three-dimensional network.

Fig. 5D further characterizes changes in protein conformation and the microenvironment surrounding tryptophan residues in the PPI gel, PPI/Ca^2+^, MPCMC/PPI, and MPCMC/PPI/Ca^2+^, as revealed by intrinsic fluorescence spectroscopy. The maximum fluorescence emission wavelength of PPI gel was 340.5 nm, which is characteristic of tryptophan residues partially located in the relatively hydrophobic interior of the protein, resulting in relatively limited active sites available for intermolecular interactions. This structural constraint may partly account for the comparatively lower gel strength, WHC, and structural stability of PPI gel. After Ca^2+^ addition, the maximum emission wavelength red-shifted to 342.1 nm, attributable to conformational unfolding of the PPI molecules that exposed tryptophan residues to a more polar microenvironment. In comparison, MPCMC appeared to induce a relatively moderate conformational change, as reflected by the maximum emission wavelength of 341.5 nm for MPCMC/PPI. MPCMC may participates in network formation through macromolecular chain entanglement, spatial filling, and non-covalent interactions. In addition, the steric hindrance may partially limit excessive protein aggregation and unfolding. The most extensive unfolding of PPI molecules and exposure of tryptophan residues were observed in MPCMC/PPI/Ca^2+^, accompanied by a further red shift of the maximum emission wavelength to 344.3 nm. In general, changes in intrinsic fluorescence intensity are collectively governed by the degree of tryptophan exposure, microenvironmental polarity, molecular aggregation state, and fluorescence quenching effects. An increase in fluorescence intensity does not necessarily indicate structural loosening of the gel network. Rather, moderate conformational unfolding is a prerequisite for the construction of an integrated gel network. The ranking of fluorescence peak intensities among the gel samples further confirmed that the coexistence of Ca^2+^ and MPCMC promoted more extensive conformational unfolding of PPI and greater exposure of fluorescent groups. These findings are also consistent with the FTIR, secondary structure, and XRD results.

### 3.6 SEM and AFM analysis

The cross-sectional micromorphology of the gel samples were observed by SEM. As shown in Fig. 6, the PPI gel, PPI/Ca^2+^, MPCMC/PPI, and MPCMC/PPI/Ca^2+^ all exhibited typical gel network structures, but differed markedly in pore size and network continuity. The PPI gel displayed a relatively loose network with large pores, and a porosity of 35.3 ± 2.7%. Single-component PPI can form a three-dimensional network through heat-induced unfolding and aggregation during gelation, the intermolecular interactions among protein molecules remain relatively limited (Chen et al., 2025; Wang, Su, et al., 2025). Such a porous and weakly connected structure is unfavorable for water immobilization and is prone to structural collapse under external stress, consistent with its lower gel strength, WHC, and rheological moduli. Previous studies have indicated that the inferior gel performance of plant proteins often originates from excessively rapid protein aggregation, which promotes the formation of large aggregates and discontinuous network structures (Wang, Su, et al., 2025; Wang, Zhang, et al., 2025). In the present study, MPCMC reduced structural defects through spatial filling and hydrophilic chain-mediated support, while Ca^2+^ enhanced network crosslinking density via ionic bridging. The synergistic action of these two components effectively restrained localized over-aggregation. Consequently, the MPCMC/PPI/Ca^2+^ developed a more homogeneous and continuous network architecture than those formed with Ca^2+^ or MPCMC alone, exhibiting a porosity of 21.2 ± 0.8%. This dense network could more effectively entrap water and restrict water migration, further corroborating the LF-NMR results.

**Fig. 6:**
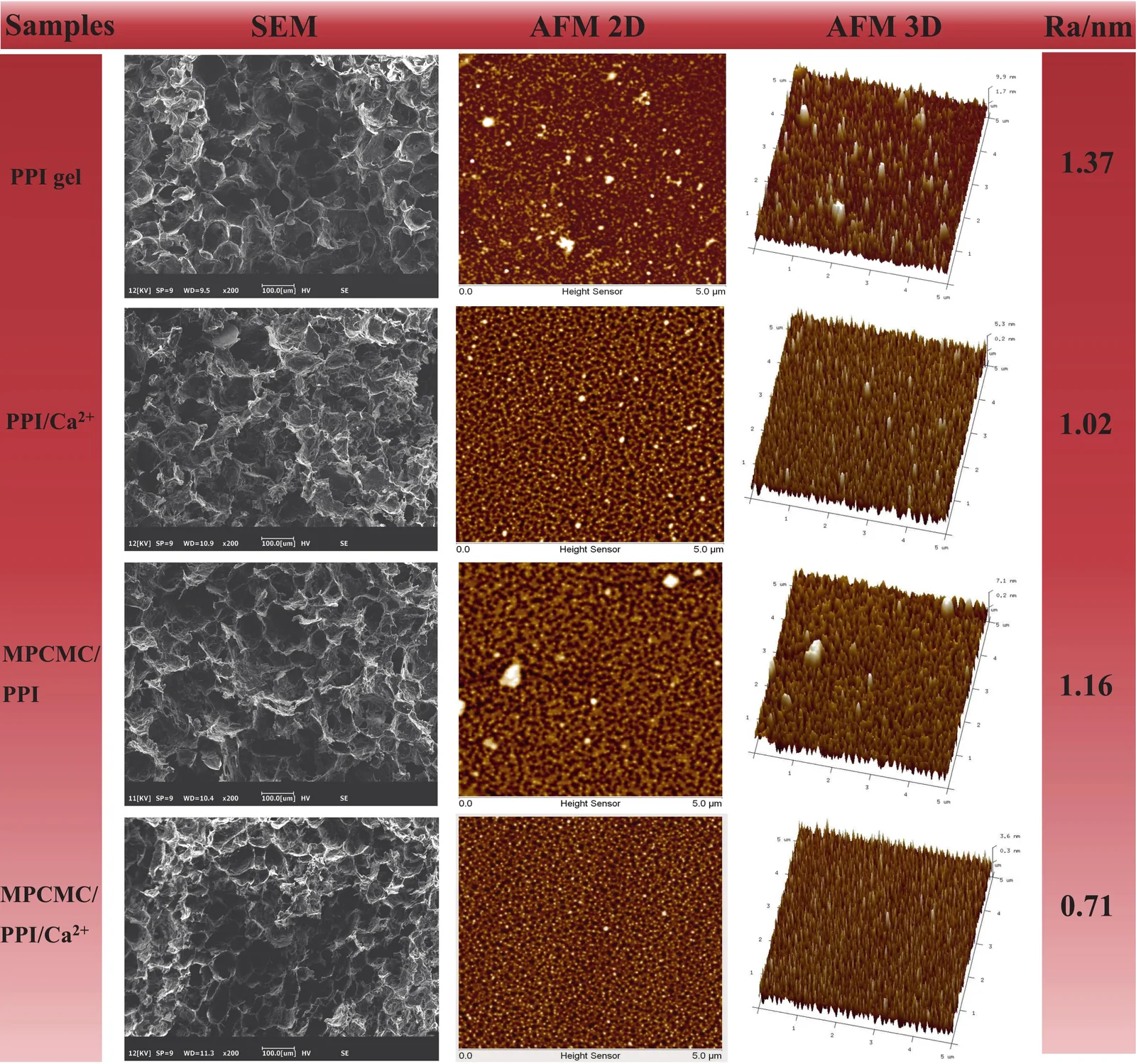
(A) SEM, and AFM images of the PPI gel, PPI/Ca^2+^, MPCMC/PPI, and MPCMC/PPI/Ca^2+^.

AFM was used to characterize the microscale surface morphology and aggregation features of the gel samples (Singh et al., 2026). The 2D and 3D AFM images (Fig. 6) showed that the PPI gel exhibited pronounced height fluctuations and heterogeneous aggregation domains, with a relatively surface roughness (Ra) value of 1.37 nm. This reflects the fact that PPI lacked sufficient cross-linking junctions and spatial support during heat-induced gelation, leading to heterogeneous protein aggregation and the formation of local structural defects. Evidently, the addition of Ca^2+^ improved the disordered arrangement of the protein, and the Ra value of PPI/Ca^2+^ decreased to 1.02 nm. Coarse protein aggregation on the gel surface was suppressed, and surface undulations were markedly attenuated. Compared with the PPI gel, MPCMC/PPI showed a lower Ra value of 1.16 nm, which may be attributed to the strong hydrophilicity of MPCMC chains and their ability to retain water and partially fill structural voids within the protein network. Nevertheless, some irregular protrusions remained on the gel surface. This phenomenon may be explained by the fact that, although Ca^2+^ or MPCMC alone can reduce gel surface roughness to a certain extent, neither is sufficient to fully suppress local protein aggregation, phase-distribution heterogeneity, or network defects. Notably, MPCMC/PPI/Ca^2+^ exhibited the lowest Ra value, only 0.71 nm. Both 2D and 3D AFM images revealed a highly smooth and uniform surface morphology, with irregular protrusions almost completely eliminated. This structural integrity is further supported by the hypothesis that Ca^2+^-mediated ionic bridging and MPCMC-driven noncovalent stabilization, which synergistically reduce local aggregation and network defects. Together, these interactions underpin the overall network stability and continuity of the gel surface structure.

### 3.7 Interaction forces, correlation analysis, and molecular simulation

Although protein solubility analysis cannot provide an absolute quantification of intermolecular forces within gel systems, it enables comparison of the relative contributions of different interactions to gel-network stabilization by evaluating the extent to which chemical solvents dissociate the gel network (Ge et al., 2026; Wan et al., 2026; Xue et al., 2025). Fig. 7A further shows the relative contributions of hydrophobic interactions, ionic bonds, hydrogen bonds, and disulfide bonds in the PPI gel, PPI/Ca^2+^, MPCMC/PPI, and MPCMC/PPI/Ca^2+^ samples. During heating, PPI molecules underwent partial unfolding, exposing hydrophobic groups originally buried within the protein interior. These exposed domains subsequently drove protein aggregation through hydrophobic association, leading to the formation of a three-dimensional gel network. Hydrogen bonds also made a substantial contribution to the stabilization of the PPI gel network. Although hydrophobic aggregation can induce PPI gelation, a network predominantly stabilized through this interaction may be more prone to heterogeneous local aggregation, enlarged pores, and limited structural continuity. Following the addition of Ca^2+^, ionic bonds became the most important stabilizing force after hydrophobic interactions. This shift indicates that Ca^2+^ promoted closer intermolecular association and aggregation of protein molecules and may reinforce network connectivity through ionic bridging. The concomitant decrease in the contribution of hydrophobic interactions does not imply weakening of the gel structure. Rather, it reflects a transition from a network dominated by hydrophobic aggregation to a composite cross-linked network jointly supported by hydrophobic interactions and ionic bridges. The incorporation of MPCMC markedly altered the interaction profile of the PPI gel, with the MPCMC/PPI network being primarily driven and stabilized by hydrogen bonding. As a hydrophilic polysaccharide, MPCMC may improve the hydration environment surrounding protein molecules and partially compensate for pores and defects in the single-component PPI network through hydration and physical filling. In addition, the pronounced decrease in disulfide-bond contribution indicates that protein aggregation relies more heavily on noncovalent interactions than on covalent cross-linking. In the MPCMC/PPI/Ca^2+^ system, ionic bonds may be the main driving force in stabilizing the gel network. The reason is that both PPI molecules and MPCMC molecular chains contain negatively charged groups capable of binding Ca^2+^, and this multi-point ionic cross-linking substantially increased the number of effective junction sites within the gel network. The presence of MPCMC does not merely compete with PPI for interaction sites; instead, it amplifies the bridging effect of Ca^2+^ while continuing to stabilize the gel skeleton through hydrogen bonding. The relative decreases in the contributions of hydrophobic interactions and hydrogen bonds further support this interpretation. Taken together, it can be inferred that the reinforcement of the PPI gel network by Ca^2+^ and MPCMC is not a simple superposition, but arises from a synergistic mechanism that promotes PPI conformational unfolding and the formation of a stable, ordered network.

**Fig. 7:**
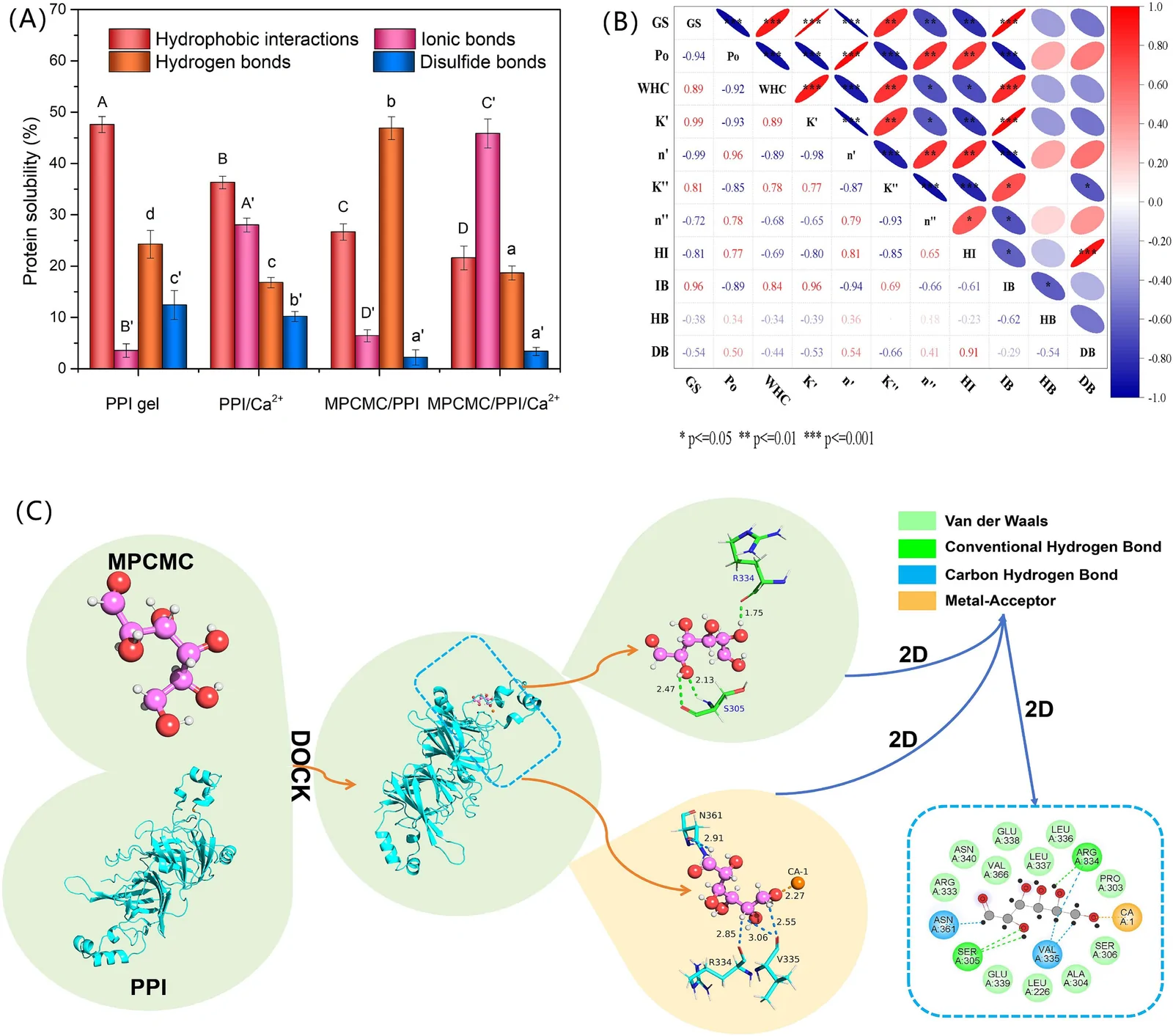
(A) Changes in intermolecular forces of the PPI gel, PPI/Ca^2+^, MPCMC/PPI, and MPCMC/PPI/Ca^2+^. (B) Correlation analysis diagram. Values with the different letter (A–D, A'–C′, a–d, a'–c') indicate significant differences (*p* < 0.05). GS represents gel strength; Po, porosity; K′, n’, K″, and n”, power-law rheological parameters; HI, hydrophobic interactions; IB, ionic bonds; HB, hydrogen bonds; DB, disulfide bonds. (C) Molecular docking diagram of MPCMC with PPI in the absence and presence of Ca^2+^.

Fig. 7B presents the Pearson correlation distribution among gel strength, porosity, WHC, power-law model parameters, and intermolecular interactions in the gel samples. Significant correlations were observed among most indicators, revealing close associations among intermolecular interactions, network structure, rheological properties, and macroscopic gel performance following the incorporation of Ca^2+^ and MPCMC. Gel strength was positively correlated with WHC (*r* = 0.89, *p* ≤ 0.001), confirming that a stronger gel network favored water immobilization. Porosity was negatively correlated with both gel strength and WHC (*r* = −0.94 to −0.92, *p* ≤ 0.001), indicating that reduced porosity provided a critical structural basis for enhanced gel strength and WHC. In terms of rheology, K′ was positively correlated with gel strength and WHC (*r* = 0.89 to 0.99, *p* ≤ 0.001), whereas n’ exhibited significant negative correlations with these parameters (*r* = −0.99 to −0.89, *p* ≤ 0.001), which is characteristic of solid-like gel behavior.

The correlations between intermolecular interactions and gel performance further provided insight into the molecular basis associated with network reinforcement. Hydrophobic interactions were negatively correlated with gel strength, WHC, and K′ (*r* = −0.81 to −0.69, *p* ≤ 0.001), whereas ionic bonds showed positive correlations with these parameters (*r* = 0.84 to 0.96, *p* ≤ 0.001). The negative correlation does not necessarily indicate that hydrophobic interactions weakened gel formation. Rather, it suggests that excessive hydrophobic aggregation may lead to local aggregation and structural heterogeneity, whereas Ca^2+^-mediated ionic bridging contributed more effectively to ordered network formation. Hydrogen bonds showed only weak correlations with most macroscopic parameters (*r* = 0.34 to 0.36, *p* > 0.05), suggesting an auxiliary role in stabilizing the cross-linked network, whereas disulfide bonds made only a limited contribution. It is precisely this correlation pattern that effectively links molecular interactions to the improvement of gel performance.

Molecular docking simulation further supported the synergistic interaction mechanism among PPI, MPCMC, and Ca^2+^. As shown in Fig. 7C, MPCMC was predicted to bind within a cavity region of PPI through multiple non-covalent interactions, including hydrogen bonding, hydrophobic interactions, van der Waals forces, and Ca^2+^-mediated coordination interactions. Specifically, MPCMC formed hydrogen bonds with Ser305 and Arg334, while additional interactions with Asn361 and Val335 contributed to complex stabilization. In addition, MPCMC coordinated with adjacent Ca^2+^ through metal-ion coordination bonds. Van der Waals and hydrophobic contacts between MPCMC and residues such as Arg333 and Pro303 further enhanced complex stability. Collectively, these multiple noncovalent interactions may increase the number of effective junction sites within the gel network, and thereby promote network densification and structural ordering.

## 4 Conclusions

This study systematically investigated the synergistic regulation of PPI gelation by MPCMC and Ca^2+^, with particular emphasis on the underlying interaction mechanisms. The results demonstrated that appropriate concentrations of MPCMC and Ca^2+^ significantly improved PPI gel performance, with the combined addition of 1.5% MPCMC and 12 mM Ca^2+^ exhibiting the optimal effect. Multiscale analyses demonstrated that MPCMC/Ca^2+^ promoted protein conformational rearrangement and transformed loose aggregates into a compact and continuous gel network. Mechanistically, Ca^2+^ served as the primary contributor to network reinforcement through ionic bridging, while MPCMC regulated intermolecular interactions via hydrogen bonding, hydrophobic interactions, and spatial filling effects, thereby facilitating network reconstruction. Overall, this study elucidates the mechanistic link between molecular interactions and enhanced gel performance by MPCMC and Ca^2+^, and provides a technical reference for the valorization of fruit pomace waste as novel green gel modifiers and the development of high-performance plant-based gels. Future studies should explore how MPCMC molecular characteristics regulate protein-polysaccharide interactions and gel network evolution, while assessing its scalability and application potential in plant-based food systems.

## CRediT authorship contribution statement

**Yuanfei Ge:** Writing – review & editing, Writing – original draft, Software, Project administration, Formal analysis, Data curation, Conceptualization. **Dan Hu:** Software, Formal analysis. **Yujie Sun:** Data curation. **Yuqing Tong:** Software. **Hanxiao Shi:** Formal analysis. **Chuyan Wang:** Supervision, Funding acquisition, Conceptualization.

## Declaration of competing interest

The authors declare that they have no known competing financial interests or personal relationships that could have appeared to influence the work reported in this paper.

## Data Availability

Data will be made available on request.
