# Supplementary material for: From agricultural waste to functional gels: Mulberry pomace-derived carboxymethyl cellulose synergistically enhances calcium-mediated peanut protein isolate gelation
Source: Food Chem X. 2026 Oct 24;40:104676. doi: 10.1016/j.fochx.2026.104676 (PMC13734912; doi:10.1016/j.fochx.2026.104676)
Supplement: Supplementary file 1 [file mmc1.docx]

**Table S1.** LF-NMR relaxation parameters of the PPI gel, PPI/Ca^2+^, MPCMC/PPI, and MPCMC/PPI/Ca^2+^.

| Samples | T_21_ (ms) | P_21_  (%) | T_22_  (ms) | P_22_ (%) | T_23_ (ms) | P_23_  (%) |
| --- | --- | --- | --- | --- | --- | --- |
| PPI gel | 0.57 ± 0.16^a^ | 3.20 ± 0.65^d^ | 87.03 ± 2.37^b^ | 94.23 ± 1.85^a^ | 2125.67 ± 158.32^d^ | 2.57 ± 0.06^b^ |
| PPI/Ca^2+^ | 1.10 ± 0.33^b^ | 2.18 ± 0.17^c^ | 102.34 ± 1.52^a^ | 96.29 ± 0.77^a^ | 1075.28 ± 47.36^b^ | 1.53 ± 0.15^a^ |
| MPCMC/PPI | 0.93 ± 0.24^ab^ | 2.81 ± 0.50^b^ | 102.34 ± 3.61^a^ | 96.37 ± 0.31^a^ | 1742.63 ± 95.74^a^ | 0.82 ± 0.03^c^ |
| MPCMC/PPI/Ca^2+^ | 0.79 ± 0.11^ab^ | 1.70 ± 0.06^a^ | 80.26 ± 1.13^b^ | 97.61 ± 0.89^a^ | 792.21 ± 75.93^c^ | 0.69 ± 0.09^c^ |

Values with the different letter (a–d) indicate significant differences (*p* < 0.05)
